# Effects of Exogenous Salicylic Acid on Drought Response and Characterization of Dehydrins in *Impatiens walleriana*

**DOI:** 10.3390/plants9111589

**Published:** 2020-11-17

**Authors:** Dragana D. Antonić, Angelina R. Subotić, Milan B. Dragićević, Danijel Pantelić, Snežana M. Milošević, Ana D. Simonović, Ivana Momčilović

**Affiliations:** Department of Plant Physiology, Institute for Biological Research “Siniša Stanković”—National Institute of Republic of Serbia, University of Belgrade, Bulevar despota Stefana 142, 11060 Belgrade, Serbia; heroina@ibiss.bg.ac.rs (A.R.S.); mdragicevic@ibiss.bg.ac.rs (M.B.D.); danijel.pantelic@ibiss.bg.ac.rs (D.P.); snezana@ibiss.bg.ac.rs (S.M.M.); ana.simonovic@ibiss.bg.ac.rs (A.D.S.); ivana.momcilovic@ibiss.bg.ac.rs (I.M.)

**Keywords:** antioxidative enzymes, catalase, flowering, malondialdehyde, peroxidase, proline, superoxide dismutase

## Abstract

*Impatiens walleriana* is a valued ornamental plant sensitive to drought stress. We investigated whether the foliar application of 2mM salicylic acid (SA) can protect potted *I. walleriana* plants from drought stress. The plants were divided into: watered plants, drought-stressed plants, watered plants treated with SA and drought-stressed plants treated with SA. The number of flowers and flower buds, relative water content (RWC), contents of malondialdehyde (MDA) and proline (Pro) and the activities of superoxide dismutases, catalases and peroxidases were recorded at different time points. Three dehydrin sequences were identified in de novo assembled leaf transcriptome: *IwDhn1, IwDhn2.1* and *IwDhn2.2.* Drought stress caused wilting, floral abortion, reduction of RWC and increased MDA—an indicator of lipid peroxidation. In response to drought, *Impatiens* accumulated Pro and induced chloroplastic Cu/ZnSOD and two peroxidase isoforms. The most remarkable drought response was strong induction of *IwDhn2.1* and *IwDhn2.2.* Rehydration restored RWC, Pro level, Cu/ZnSOD activity and dehydrins expression in drought-stressed plants approximately to the values of watered plants.SA had ameliorating effects on plants exposed to drought, including prevention of wilting, preservation of RWC, increased Pro accumulation, modulation of antioxidative activities and remarkable decrease of lipid peroxidation, but without effects on flowers’ preservation.

## 1. Introduction

The genus *Impatiens* (Balsaminaceae) is one of the largest angiosperm genera with more than 1000 species of annual or perennial herbs [1,2]. The *Impatiens* species are primarily distributed in the Old World tropics and subtropics, growing mainly in mesic or wet conditions [2]. Zygomorphic, 5-merous flowers, diverse in corolla morphology and colors, characterize the genus [1]. Due to their beauty and long flowering period, many *Impatiens* species are of great horticultural value and are cultivated worldwide as bedding or potted plants. Among them, *Impatiens walleriana* Hook. f. (‘Busy Lizzie’), an eastern African native, is the most popular. *I. walleriana* has fleshy, succulent leaves and stems and a variety of flower colors.

One of the major obstacles in *I. walleriana* production, transport and sale display is the plants’ tendency to wilt quickly when exposed to drought [3,4,5]. Drought is one of the most adverse stresses, which triggers morphological, physiological and biochemical changes in plants, including a reduction of growth, gas exchange, photosynthesis and respiration [6,7].Common consequences of drought are the reduction of relative water content (RWC), leaf water potential, transpiration rate, stomatal conductance and, consequently, decrease in CO_2_ assimilation, inhibition of photosynthesis and shifts in C and N metabolism [7,8].Like all other stresses, drought leads to oxidative stress caused by an imbalance between production of reactive oxygen species (ROS) and the capacity of enzymatic and non-enzymatic antioxidative defense systems to process ROS [6].Impairment of electron transport chains in chloroplasts, mitochondria and other cellular compartments, caused by water stress, results in accumulation of superoxide anion radicals (O_2_^•-^), hydroxyl radicals (•OH), hydrogen peroxide (H_2_O_2_), and singlet oxygen (^1^O_2_); the accumulated ROS may react with proteins, membrane lipids, DNA and other cellular constituents causing oxidative damage [6,9]. The extent of ROS accumulation and membrane damage is commonly evaluated by measuring the content of malondialdehyde (MDA)—one of the products of membrane lipids peroxidation [7,10,11].

Physiological mechanisms of plants’ drought resistance include osmotic adjustment, activation of enzymatic and non-enzymatic antioxidative systems and stabilization of cell membranes [6]. Plant cells can maintain turgor, to some extent, by decreasing their osmotic potential through the accumulation of compatible solutes (osmolytes), primarily proline (Pro) [6,12]. Proline is not only involved in the osmotic adjustment, but also in cell membranes’ stabilization and ROS scavenging [12].

Antioxidative enzymes that control ROS level in cells—superoxide dismutase (SOD), catalase (CAT), and peroxidase (POX)—are commonly induced in response to drought. SODs (EC 1.15.1.1) protect plant cells under both physiological and pathological conditions against the deleterious effect of superoxide anion radical (O_2_^•-^), by converting it into H_2_O_2_ and O_2_, so their activity is an important indicator of the antioxidative status of stressed tissues. Considering the impermeability of cell membranes to charged O_2_^•-^, SODs must be present at all cellular compartments where O_2_^•-^ is generated [13]. Thus, FeSODs are present in plastids, MnSODs in the mitochondrial matrix, peroxisomes and also in the cell wall, while Cu/ZnSODs are found in the cytosol, peroxisomes, plastids, and possibly extracellular space [13,14].Catalases (E.C. 1.11.1.6) are peroxisomal tetrameric heme-containing enzymes capable of dismutating hydrogen peroxideinto water and oxygen [15]. CATs are encoded by only three genes in plant species analyzed to date, with relatively specific roles in controlling H_2_O_2_ levels produced through various metabolic pathways [15].

Class III peroxidases (POX; E.C. 1.11.1.7) are ubiquitous in higher plants, where they are present as large gene families. POXs are generally secreted into the cell wall, external medium and the vacuole [16]. Depending on reaction conditions and isoform specificity, POXs can be involved not only in a regular peroxidative cycle, where they consume hydrogen peroxide to oxidize phenolic compounds, lignin precursors or other secondary metabolites, but also in a recently described hydroxylic cycle, which leads to the formation of various ROS [16,17]. The POX-catalyzed reactions occur in the apoplast and are important for a diverse range of physiological and developmental processes, with apparent functional specialization of the isoforms [16,17].Even though POXs are commonly considered as enzymes involved in desiccation tolerance [16,17], their specific roles during drought response are unclear and rarely connected to specific genes or substrates. Generally, POXs contribute to scavenging of SOD-generated H_2_O_2_, but probably also in adjustments of cell wall elasticity following dehydration/rehydration of the plant tissues. CATs are responsible not only for removal of H_2_O_2_ produced by SOD activities in chloroplasts and other cellular compartments, but also for detoxification of H_2_O_2_ produced during photorespiratory oxidation of glycolate, which is enhanced when photosynthesis is impaired [6]. Coordinated regulation of SOD, POX and CAT activities, along with other antioxidative enzymes, is crucial for maintaining homeostasis during drought stress and recovery.

Dehydrins belong to late embryogenesis abundant (LEA) proteins (group 2) and are commonly induced in response to drought and other abiotic stresses [8,18]. Experiments in vitro suggest that dehydrins can stabilize membranes, protect proteins from aggregation, cryoprotect enzymes, protect nucleic acids, scavenge ROS and can bind small ligands including water, ice crystals and metal ions, but these findings need the in vivo conformation [18,19,20]. Having mostly polar and charged residues, dehydrins lack defined secondary and tertiary structure, and are thus defined as intrinsically disordered proteins (IDPs). The dehydrin family is characterized by the presence of at least one Lys-rich K-segment and may have other conserved functional segments [18]. At the cellular level dehydrins are commonly located in the cytoplasm, associated with the plasma membranes and in the nucleus, while at the plant level they are predominantly localized in cells surrounding xylem vessels, in the apical meristem and root tips [8,18].

Agricultural and horticultural drought stress management includes exogenous application of natural or synthetic growth regulators that may increase drought tolerance in certain species: abscisic acid, gibberellic acid, 1-aminocyclopropane-1-carboxylic acid (ethylene precursor), uniconazole (inhibitor of gibberellin biosynthesis), brassinolide, jasmonates, benzyladenine and salicylic acid (SA) [6]. Salicylic acid is anubiquitous and potent phytohormone involved in diverse physiological and developmental processes, including responses to biotic and abiotic stresses [21,22]. SA applied in low concentrations generally has an acclimation-like effect, leading to enhanced tolerance toward different abiotic stresses, primarily due to increased antioxidative capacity of the plant tissues [21,23]. Exogenous SA can also improve growth under water deficit conditions in a number of species [9,11,24,25]. Plant responses to exogenous SA depend on the species, developmental stage, the applied concentration, mode of application (foliar spraying, seed soaking, stem injection, irrigation, addition to culture media or hydroponic solution) and the endogenous SA level [22,23].

We have previously described morphological, physiological and biochemical responses of in vitro-grown *I. walleriana* to prolonged polyethylene glycol (PEG)-induced physiological drought [25]. PEG (1–3%)reduced growth, fresh weight, the number of developed leaves and shoots, RWC and chlorophyll content, increased leaf water loss and SOD, CAT and POX activities and caused accumulationof proline, H_2_O_2_, and MDA [25]. In this experimental system exogenous SA (1–3 mM added to the medium) counteracted the effects of PEG on growth, physiological and biochemical parameters, except on proline accumulation, which was enhanced by both PEG and SA [25]. These in vitro studies have the advantages of stringent control of the physical environment and nutrient supply, aseptic conditions and isolation of a single factor—physiological drought—from other stresses that are commonly experienced by potted or field-grown plants. However, the true potential of SA application in ameliorating drought stress, particularly regarding flowering as the most important *I. walleriana* feature, can be better evaluated by studying ex vitro-grown plants exposed to drought. In addition, ex vitro- and in vitro-grown plants may significantly differ in expression and/or activity of antioxidative enzymes [14]. Here, we present the effects of drought and SA on morphological, physiological and biochemical parameters of potted *I. walleriana.* The study has several aims: (1) to investigate the potential of SA on protecting this popular, horticultural species in nurseries from drought stress; (2) to test whether exogenous SA can preserve flowering in drought-stressed plants; (3) to compare drought responses of ex vitro-grown plants to previously reported results for in vitro-cultivated plants; and 4) to evaluate the role of dehydrins by analyzing their structure and expression in response to drought and SA.

## 2. Materials and Methods 

### 2.1. Plant Materials and Growth Conditions

*Impatiens walleriana* variety Accent Premium Red (Syngenta Flowers North America, Gilroy, CA, USA) was used in the experiment. Seeds were planted in 3 L pots (one seed per pot) filed with well-moistened commercial substrate Floradur B Pot Medium-Coarse (Floragard, Oldenburg, Germany) and covered with cling wrap to assure high humidity. After seed germination (3–4 days), the pots were regularly vented a few h every day to ensure seedling acclimation to 70% humidity, and plastic wrap was removed after one week. Seeds were germinated and plants were grown under controlled conditions (20/18 °C, day/night temperature, 16 h photoperiod, 70% humidity, light flux 140 μmol m^−2^s^−1^) in the plant growth chamber Fitoclima PLH 1200 (Aralab, Rio de Mouro, Portugal).

### 2.2. Experimental Design

**Figure 1:**
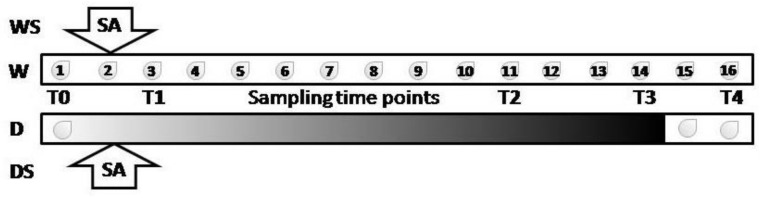
Experimental scheme. Droplets represent days of the experiment when the plants were watered. Drought stress severity is indicated by dark shading. Arrows indicate that salicylic acid (SA) was applied on the 2nd day of the experiment. T0 trough T4 are sampling times. Watered plants (W); drought-stressed plants (D); watered plants treated with SA (WS); drought-stressed plants treated with SA (DS).

At the beginning of the experiment, eight-week-old *I. walleriana* plants were watered until the soil substrate was fully saturated. Plants were divided into four experimental groups with 12 plants per group. The first (W) and second (WS) groups were watered during the entire experiment with 75 mL of water every day, while the third (D) and fourth (DS) groups were water-deprived until the 14th day of the experiment. On the 2nd day of the experiment, leaves of WS and DS plants were sprayed with 2 mM SA, while leaves of W and D plants were sprayed with distilled water. On the 15th day of the experiment, plants from all groups were watered, and thus the rehydration was carried out in groups D and DS. Plant material (leaves) was sampled on: the 1st day of the experiment, after the plants were watered (T0); 3rd day—24 h after SA application in WS and DS groups (T1); 11th day of the experiment, when first symptoms of drought were visible in D group (T2); 14th day, when drought effect was extremely morphologically noticeable in D group (T3); and on the16th day of the experiment—24 h after rehydration (T4). The timeline of the experiment is schematically presented for clarity (Figure 1).

### 2.3. Measurement of Relative Water Content (RWC), Malondialdehyde (MDA) and Proline (Pro)Contents

Ten leaf discs (5 mm in diameter) were sampled from plants of four experimental groups at each sampling time point (T0–T4). RWC was determined using the formula RWC (%) = (FW−DW)/(TW−DW) × 100 [26], where FW is fresh weight, DW is dry weight of 10 leaf discs after lyophilisation, TW is turgor weight after 24 h hydration in water. The assay was performed with four replicas per each experimental group and sampling time point. Free MDA and Pro content were quantified as described by [25].

### 2.4. Isolation of Leaf Proteins and In-Gel Enzymatic Activities

Extraction of crude soluble proteins from *Impatiens* leaves, separation of POX (100 μg protein/lane) and CAT isoforms (35 μg protein/lane), as well as POX and CAT in-gel assays, were performed as described by [27]. The activity of SODs was examined by a modified method of [28] as follows. Total leaf soluble proteins (40 μg) were separated by nativepolyacrylamide gel electrophoresis (PAGE) on 10% resolving gel at 4 °C. Gels were incubated in the reaction mixture (0.1 mMnitroblue tetrazolium, 0.1 M EDTA, 0.03 mM riboflavin, and 2 mM N,N,N’,N’-tetramethylethylenediamine in 0.1 M K-phosphate buffer, pH 7.8) for 30 min in the dark, rinsed in distilled water and illuminated until the bands became apparent. To discriminate the isoforms, the assays are performed in the presence or absence of inhibitors H_2_O_2_ and KCN, whereby Cu/ZnSOD is sensitive to both inhibitors, FeSOD is inactivated by H_2_O_2_ but resistant to KCN inhibition, while MnSOD is neither inhibited by KCN nor inactivated by H_2_O_2_ [13]. The gels with separated SOD isoforms were incubated in the inhibitor solutions (1M KCN or 5 mM H_2_O_2_) 30 min before staining [14].

### 2.5. Immunoblot Analysis

For native PAGE immunoblotting, 30 μg of leaf proteins were loaded on 10% polyacrylamide gels and separated at 100 V for 2.5 h at 4 °C using Mini-PROTEAN Tetra Cell System (Bio-Rad, Hercules, CA, USA). Following PAGE, proteins were transferred using the same system (at 200 mA for 45 min to a polyvinylidene difluoride (PVDF) membrane (Bio-Rad, Hercules, CA, USA)) and blots were processed as described by Momčilović and Ristić [29]. Blots were probed with anti-chloroplastic Cu/ZnSOD (raised against *Arabidopsis thaliana* Cu/ZnSOD recombinant protein) antibodies (Agrisera AB, Vännäs, Sweden).

### 2.6. RNA Isolation

Total RNA for RNA sequencing was isolated from leaves of unstressed 12-week-old plants. For quantitative real-time polymerase chain reaction (RT-qPCR) analyses, leaves from W, WS, D and DS plants were sampled at T0-T4 time points. Since *I. walleriana* leaves are flashy, with relatively low RNA content and rich in polyphenolics and polysaccharides, several protocols for RNA isolation were compared [30] and the optimal procedure of Gasic et al. [31] was used. The RNA samples were treated with DNase I (Thermo Fisher Scientific Baltics UAB, Vilnius, Lithuania) to remove DNA contamination. The concentration and purity of the RNA samples were measured using Nano Photometer N60 (Implen, Munich, Germany).

### 2.7. RNA Sequencing, De Novo Transcriptome Assembly and Functional Annotation

Ultra-high throughput RNA sequencing was performed on HiSeq 2500 Illumina platform in standard paired-end mode by Genomix4Life (Salerno, Italy). Quality control of the raw sequence data was performed using FastQC [32] while the tool Cutadapt [33] was used to remove the adapter sequences. The high-quality reads were used as input to perform de novotranscriptome assembly using Trinity [34]. Candidate coding regions were identified and translated into proteins (at least 100 amino acids long) using TransDecoder [34]. Functional annotation was performed using Trinotate, a comprehensive suite which combines homology search to known sequence data (BLAST+/SwissProt), protein domain identification (HMMER/PFAM), protein signal peptide and transmembrane domain prediction (signalP/tmHMM), and leveraging various annotation databases (eggNOG/GO/Kegg databases). The main features of the obtained transcriptome are given in Table S1.

To identify dehydrin sequences in *I. walleriana* leaf transcriptome, in addition to functional annotation, six-frame translation of all the transcripts corresponding to min 100 amino acids was used to search for the PF00257.18 hmm model (dehydrins) using hmmsearch function in hmmer 3.1b2 [35]. Four dehydrin sequences were identified, but one failed to produce PCR amplicons of the expected size and was discarded from further analyses.

### 2.8. Quantitative Real-Time Polymerase Chain Reaction (RT-qPCR)

Reverse transcription (RT) of RNA samples was carried out using RevertAid First Strand cDNA Synthesis Kit (Thermo Fisher Scientific Baltics UAB, Vilnius, Lithuania) with oligo-dT primers, according to the manufacturer’s protocol. Standards for absolute qPCR quantification were prepared from PCR products extracted from the agarose gel using GeneJET Gel Extraction Kit (Thermo Fisher Scientific Baltics UAB, Vilnius, Lithuania) which were serially diluted. qPCR reactions (10 μL) contained 1 μL of RT reaction (cDNA corresponding to 100 ng RNA or standard), 5 µL of Maxima SYBR Green/Rox qPCR Master Mix (Thermo Fisher Scientific Baltics UAB, Vilnius, Lithuania) and 0.5 μM primers (sequences given in Table S1). For each sample, qRT-PCR was performed in three biological replicates. Primer specificity was confirmed by local BLAST, electrophoretic sizing of the PCR products, and melting curve analysis. Reaction steps included initial denaturation (95 °C/5 min), followed by 36 cycles of denaturation (95 °C/30 s), annealing (at gene-specific Ta/30 s), and extension (72 °C/30 s), followed by final extension (72 °C/1 min) and melting curve analysis. Constitutive expression of the 18S rRNA gene was confirmed in parallel using universal primers [36].

### 2.9. Statistical and Bioinformatical Analyses

Statistical and bioinformatical analyses were performed using the R programming language for statistical computing [37].

Flower and bud data were analyzed using Poisson regression. Within each sampling time, a general linear Poisson model with log link was fitted with the following predictors: dehydration, SA and their interaction. These models were compared with models with fewer terms using likelihood ratio tests, and the simplest model for each time point was selected. Multiple comparisons were performed for models with significant terms (*p* < 0.05) by comparing estimated marginal means using the emmeans package. *p*-values obtained from these comparisons were adjusted jointly using the False Discovery Rate (FDR) method of Benjamini and Hochberg [38].

RWC, MDA and proline data were analyzed using three-way factorial analysis of variance (ANOVA, type I sum of squares) with dehydration, SA and time, as well as all possible interactions as categorical predictors. Model assumptions were checked by visual inspection of residual plots to verify that the data arehomoscedastic and havenormally distributed residuals. Multiple comparisons were performed by comparing estimated marginal means within each sampling time point using Tukey’s method for multiple comparisons as implemented in the emmeans package [39].

Dehydrin expression data were analyzed using the ΔΔCt method [40], using WS at T0 as a control sample. The 18S rRNA gene was amplified using universal primers [36], which showed constitutive expression, and was used for normalization. For genes with detectable expression in the majority of treatments, the ΔCt values were statistically compared using three-way factorial ANOVA (type I sum of squares) with dehydration, SA and time, as well as all possible interactions as categorical predictors. Models were simplified by the removal of insignificant terms as determined by F-tests starting from the most complex interaction. Model assumptions were checked by visual inspection of residual plots to verify that the data is homoscedastic and has normally distributed residuals. Where appropriate, multiple comparisons were performed by comparing estimated marginal means within each sampling time point using Tukey’s method for multiple comparisons as implemented in theemmeans package [39]. For sparsely expressed genes, expression levels between treatments were compared using Welch’s *t*-test [41].

Dehydrin proteins architecture was schematically presented using ragp R package [42] which utilizes hmmscan (https://www.ebi.ac.uk/Tools/hmmer/search/hmmscan) predictions to infer positions of domains, and Espritz web server [43] predictions to infer positions of disordered regions. Dehydrin specific motifs were identified using regular expression search in R or by using pattern matching via the Biostrings package [44].

## 3. Results and Discussion

### 3.1. Effect of Drought Stress and Salicylic Acid (SA) on Flowering of Potted ImpatiensWalleriana Plants

Potted ornamentals are frequently exposed to severe water deficit at point of sale, which has adverse effects on flowering and their aesthetic value [3,45]. In our previous work with in vitro-grown *I. walleriana,* we have shown that SA ameliorated the effects of physiological drought [25]. However, flowering, as the most important commercial value of *Impatiens*, could not be evaluated in the in vitro setup. Here we investigate the effects of SA applied as 2 mM foliar spray on flowering of watered and drought-stressed potted *Impatiens* plants.

**Figure 2:**
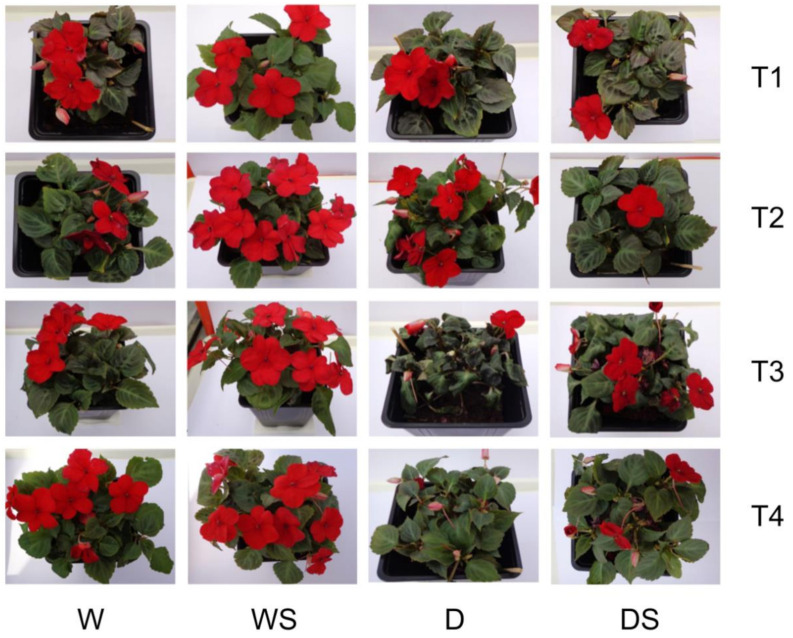
Appearance of the four test groups of plants at different time points. watered plants (W); drought-stressed plants (D); watered plants treated with salicylic acid (SA) (WS); drought-stressed plants treated with SA (DS). T1–T4 are different time points as indicated in Figure 1.

Visual observation of the four groups of plants at different time points suggests that in control W plants the number of open flowers is somewhat higher at later time points (T3, T4) than at the beginning of the experiment, in contrast to stressed D plants, where drought stress (T3) clearly reduces the number of flowers (Figure 2). D plants after ten days of drought (T2) had a similar appearance as unstressed W plants, but after 13 days of drought (T3), they wilted and shed most of their flowers. Application of SA apparently had no effect on watered plants, and had no effect of flower preservation under drought, but it protected drought-stressed plants from wilting (compare D and DS at T3). Both D and DS plants responded well to rehydration (T4, Figure 2).

**Figure 3:**
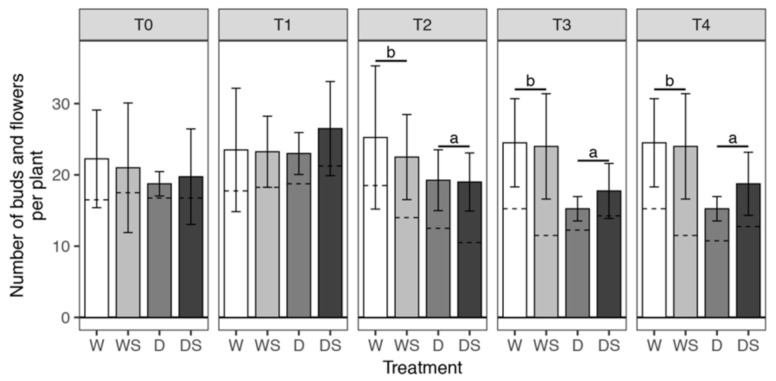
Average number of flower buds and flowers developed per plant. Error bars represent standard deviation of four biological replicates (four plants) T0–T4 are different sampling points (see Figure 1). watered plants (W); drought-stressed plants (D); watered plants treated with SA (WS); drought-stressed plants treated with SA (DS). The average number of flower buds is indicated by the lower part of the bars, below the dashed lines, while the upper part of the bars corresponds to the average number of open flowers. The total number of buds and flowers was modeled within each sampling time (T0–T4) using Poisson regression with log link. Dehydration was shown to have a statistically significant effect at T2, T3 and T4 (Table S2). Pairwise comparisons of estimated marginal means were conducted at these time points for the effect of dehydration (W + WS group compared to the D + DS group); *p*-values obtained from these comparisons were adjusted jointly using the FDR method and significant differences between groups (*p* < 0.05) are shown with a compact letter displayed above the bars.

Statistical analysis confirmed that SA application has no effect on flowering, and that the only factor that affects the number of flowers including buds, in our experimental setup, is drought stress (Figure 3 and Table S2). Drought stress significantly reduced the number of flowers and flower buds in D and DS groups as compared to watered W and WS plants at T2 and T3 (Figure 3). Even though rewatering (T4) had an immediate effect on leafage appearance in the D group (Figure 2), it had no effect on the number of flowers and flower buds in any group (T3 and T4, Figure 3), probably because the reappearance of the flowers requires more time.

Several authors have also reported a reduction of flower number in potted *I. walleriana* exposed to drought in different experimental setups [4,5,45,46]. However, none of these reports addressed the mechanism causing a decrease in floral numbers in drought-stressed *Impatiens*; generally, these mechanisms differ among different species and may include disturbed floral initiation, floral abortion, or a decrease in plant size resulting in fewer locations for flower initiation [4]. Our results show that severely stressed D and DS plants had a significantly lower number of flowers and buds than watered plants of the same age (T3–T4, Figure 3), but it is also obvious that the numbers of flower buds at these time points are similar in all groups, while the number of open flowers decreased due to stress (Figure S1, Table S3 and Table S4). These findings suggest that the mechanism of flower number reduction in *Impatiens* is floral abortion, whereas floral initiation remains undisturbed. Reduction of flowering is a defense mechanism, because plants subjected to various stresses, particularly drought, reduce flowering to save assimilates needed for survival [45].

In a similar setup with potted *I. walleriana* exposed to drought for 2–12 days, as water withholding duration increased, the average number of open flowers per plant showed a decreasing trend [46]. The authors showed that plants treated with abscisic acid (ABA) displayed fewer moisture stress symptoms, but ABA had no effect on the number of flowers, just like SA in our experiment. However, in many other species SA had prominent effects on flowering induction, e.g. in *Lemna*, soybean and *Sinningia speciosa,* while in others, such as *Spirodela* and *Wolfia microscopica,* acetylsalicylic acid had similar effects [21].

### 3.2. Effect of SA on RWC, MDA and Pro content of Drought-Stressed Impatiens Plants

**Figure 4:**
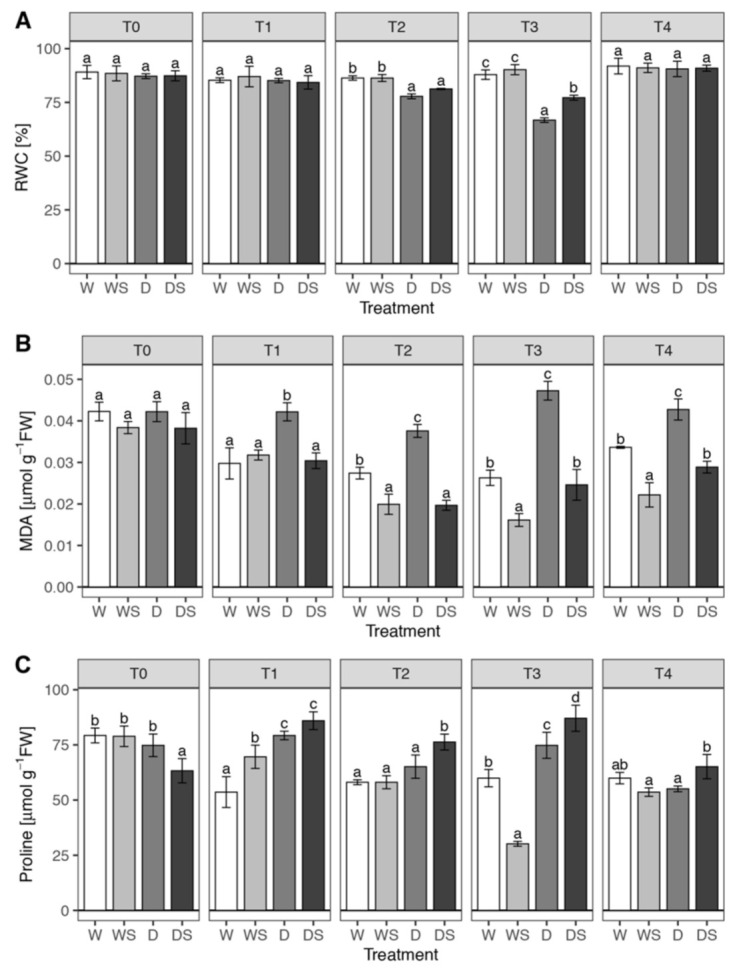
Changes in relative water content (RWC) (**A**), malondialdehyde (MDA) (**B**), and Proline (**C**) in *Impatiens* plants under drought stress and in response to SA. Watered plants (W); drought-stressed plants (D); watered plants treated with SA (WS); drought-stressed plants treated with SA (DS). T0–T4 are different sampling points (see Figure 1). Each bar represents the average RWC, MDA or proline of three biological replicates. Variation between biological replicates is indicated by error bars representing standard deviation. The data was analyzed using factorial analysis of variance (ANOVA, Tables S5, S6 and S7). The effect of the three-way interaction dehydration:SA:time was significant on each dependent variable, so pairwise comparisons of estimated marginal means were conducted within each sampling time (T0–T4) using Tukey’s method and significant differences (*p* < 0.05) are indicated by a compact letter displayed above the bars.

Reduction of RWC is one of the most common consequences of drought stress, reported for virtually all plants tested [6,7]. Since RWC reflects plant water status and is related to dehydration tolerance [7], it is commonly evaluated in drought-related studies. Factorial ANOVA showed a significant three-way interaction of dehydration: SA:time (Table S5) indicating that the effect of SA differs between watered and stressed plants at some time points. Well-watered *Impatiens* plants at the beginning of the experiment (T0) had RWC above 87% (87.2–89.1%), which did not change during the course of the experiment in W and WS groups (Figure 4A). Drought-stressed (D) plants had significantly reduced RWC after 10 days of water deprivation (77.8% at T2) as compared to W plants, while severely stressed plants had RWC as low as 66.8% after 13 days of drought (T3, Figure 4A). The protective effect of SA was significant after 13 days of drought, where DS plants had on average 10.4% higher RWC as compared to D plants (T3, Figure 4A). Both D and DS plants completely restored RWC upon rewatering (T4) to the initial (control) level.

The recorded RWC for severely stressed *Impatiens* plants of 66.8% is lower than reported ”lethal RWC“ for this species of 73%, where the lethal point was defined as a stage when fewer than eight live leaves remained on a plant [3]. Considering the good response to rehydration of the stressed plants (T4, Figure 2 and Figure 4A), the actual lethal point, obviously, has not been reached in our experimental system after 13 days of water deprivation. Augé at al. [3] reported that it takes 55 days of water deprivation for potted *I. walleriana* to reach the lethal point. Chyliński et al. [45] reported an even larger drop of RWC in *I. walleriana*, where unstressed plants had RWC of 96.4%, while in severely stressed plants it dropped to only 56.1%. Our previous experiments with in vitro-grown *I. walleriana* under PEG-imposed physiological drought also showed a decrease of RWC of *Impatiens* shoots with increasing PEG content, up to 38% reduction at 3% PEG in comparison to the control [25]. It is obvious that the drop of RWC under water stress depends on the experimental conditions, but it is often larger in *I. walleriana* when compared to other species [45]. The performance of *I. walleriana* under water stress as compared to other plant species is best illustrated by the facts that *I. walleriana* has been ranked 3rd most dehydration intolerant species among 30 herbaceous or woody ornamentals tested [3] and performed poorest among 17 herbaceous annual ornamentals in a field experiment with reduced irrigation [47]. This is understandable knowing that *Impatiens*, coming from tropical forests, is very succulent, with leaves lacking any serious protection against water loss during water stress [45].

The ameliorating effect of exogenous SA on RWC preservation under drought stress has been reported for many species. For instance, higher RWC in SA-pretreated plants in comparison to untreated controls has been shown in wheat [48], *Celosia argentea* [24], mustard [11] and rice [49] exposed to drought, while in *Ctenanthe setosa* even 1 µM SA was effective in partial RWC preservation after prolonged drought [10]. It is interesting that SA increased RWC in drought-stressed plants even when applied after the drought exposure [9]. On the other hand, SA had little or no effect on RWC in unstressed plants [11,24,25], except in tomato [9].

MDA is one of the products of membrane lipids peroxidation, so it is often quantified as an indicator of membrane damage under oxidative stress [7]. Factorial ANOVA showed a significant three-way interaction of dehydration: SA: time (Table S6) indicating that watered and plants under drought stress respond differently to SA at some sampling time points. The content of MDA in unstressed (W) plants slightly decreased during the course of the experiment (even though this was not statistically tested, Figure 4B). Plants exposed to drought stress (D) accumulated significantly more MDA in comparison to W group at all time points beyond T0, but this difference was most prominent in severely stressed plants (80% at T3). Rehydration (T4) caused a drop in MDA content in the D group, but not to the control (W) level. SA application affected the unstressed plants, since WS plants had lower MDA content in comparison to W plants at T2–T4 time points, up to 39% reduction at T3 (Figure 4B). SA application completely protected membranes in plants exposed to drought, since the level of MDA in DS plants was at the control (W) level or lower at all time points. In the in vitro-grown *I. walleriana*, the application of 2 mM SA also completely protected PEG-treated plants from MDA accumulation (or membrane damage), only in this case, SA had no effect on unstressed plants [25]. This difference could be because of the different physiological status of ex vitro-grown plants.

Increased membrane lipids peroxidation in plants exposed to drought and membrane protective effect of SA has been reported in many species and experimental systems. In *Ctenanthe setosa* plants treated with SA, MDA content significantly decreased during the drought period, while it increased in control leaves [11]. Similar effects of SA were obtained with *Celosia argentea* [24], *Brassica juncea* [11], rice [49] and wheat, where SA also had a positive effect on membrane stability index [48]. In tomato plants exposed to drought and subsequently treated with SA, SA caused a remarkable decrease in electrolyte leakage and lipid peroxidation and a significant increase in the membrane stability index [9].

Accumulation of Pro is the first response of plants exposed to water-deficit stress [7], so we investigated the changes in Pro level in the four plant groups during the course of the experiment. Statistical analysis of proline content (Table S7) indicated a significant three-way interaction of dehydration: SA:time. Pro content in watered plants was somewhat lower in T1–T4 time points in comparison to the start of the experiment (Figure 4C). Pro accumulated in D group more than in W control during the exposure to drought (with a statistically significant difference at T1 and T3), but it decreased to the control level upon rehydration at T4. The application of SA had minimal effect on watered plants (with the exception of WS at T3, which represents a clear outlier), but it caused a significant accumulation of Pro in D plants (Figure 4C). Positive effects of both water stress and SA on Pro accumulation were also apparent in *I. walleriana* exposed to PEG in vitro [25].

The combined effect of drought and SA on Pro level differs depending on species (and probably also on experimental conditions). In some species, both drought and SA increase Pro accumulation, just like in *Impatiens*; this is the case with *Brassica napus*, where SA pretreatment increased Pro content in both unstressed and drought-stressed plants [50], and with tomato, where SA post-treatment had the same effect [9]. In the case of *B. napus*, it was shown that SA induced the expression of genes involved in Pro synthesis (two pyrroline-5-carboxylate synthetases and pyrroline-5-carboxylate reductase) and reduced expression of genes involved in Pro degradation (proline dehydrogenase and pyrroline-5-carboxylate dehydrogenase) [49]. By contrast, while drought stress caused a profound increase in Pro content in mustard seedlings, SA supplemented stressed seedlings maintained significantly lower Pro content [11]; a similar response to drought and SA was reported for rice grown either in soil or in hydroponics [49].

Proline is not only an excellent compatible solute involved in osmotic adjustment, but a molecule with many protective roles in stressed cells, including ROS detoxification, metal chelation, stabilization of membranes (thereby preventing electrolyte leakage), stabilization of proteins and mitochondrial electron transport complex II, storage of carbon and nitrogen for use after relief of water deficit and trigger for specific gene expression, which can be essential for plant recovery from stress [6,7,12]. Thus, the drought-ameliorating SA effect in *Impatiens* is at least in part due to the stimulation of Pro accumulation in stressed plants.The accumulated Pro could help in RWC maintenance (Figure 4A) through osmotic adjustment. However, comparison of MDA and Pro contents (Figure 4B,C) suggests that Pro accumulation resulting from drought signaling (in D group) apparently does not protect membranes from oxidative damage effectively, because D group has the highest MDA levels at all time points. On the other hand, WS plants which have the lowest MDA content at T2–T4 time points, do not have higher Pro levels than other groups. It can only be concluded that in *Impatiens* Pro accumulation does not protect cell membranes efficiently and that membrane-protective effects of SA apparently rely on mechanisms unrelated to Pro accumulation.For these reasons, other possible mechanisms of SA-mediated protection of stressed plants are further explored, particularly SA effects on antioxidative enzymes.

### 3.3. Effect of SA on Antioxidative Enzymes Activities under Drought Stress

Although SA may cause oxidative stress to plants, partially through a transient accumulation of hydrogen peroxide, when applied at suitable (usually low) concentrations, SA was found to enhance the efficiency of antioxidant system in plants [21,23]. Here we investigated the effects of 2 mM SA, a concentration effective in ameliorating PEG-imposed drought in in vitro-grown *I. walleriana* [25] on SOD, CAT and POX activities in watered and drought-stressed *I. walleriana* plants.

**Figure 5:**
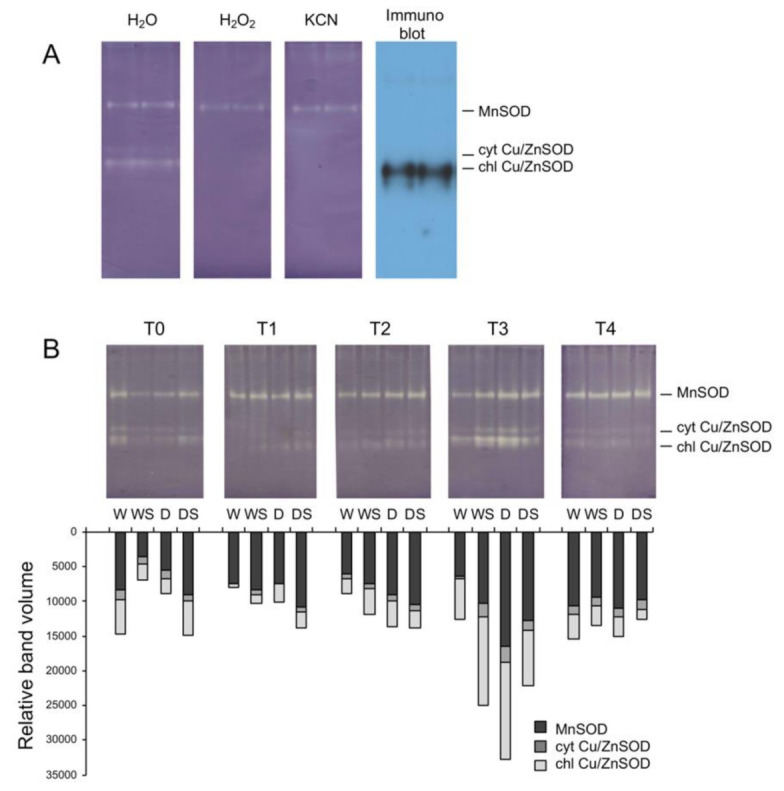
(**A**)—Identification of superoxide dismutase (SOD) isoforms after native polyacrylamide gel electrophoresis (PAGE) separation. SOD was assayed in the presence or absence of inhibitors 1M KCN or 5 mM H2O2; the proteins separated by native PAGE were also immunoblotted using anti-chloroplastic Cu/ZnSOD antibodies. (**B**)—Zymograms of SOD isoforms. Total soluble proteins isolated from leaves (40 μg proteins/lane) of watered plants (W), drought-stressed plants (D), watered plants treated with SA (WS) or drought-stressed plants treated with SA (DS) were separated by native PAGE and assayed for SOD activity. T0–T4 are different sampling points (see Figure 1). Relative band volumes for individual SOD isoforms are represented by stacked bars.

In *I. walleriana* leaves, three SOD isoforms were detected by native PAGE separation: one MnSOD, insensitive to both KCN and H_2_O_2_, and two Cu/ZnSODs which were sensitive to both inhibitors (Figure 5A). Immunoblotting of proteins separated by native PAGE with anti-chloroplastic Cu/ZnSOD antibodies confirmed that the fastest migrating band was chloroplastic Cu/ZnSOD, while the other Cu/ZnSOD band was predicted to be cytosolic Cu/ZnSOD isoform which is also broadly distributed in plants [13]. SOD zymograms of proteins isolated from in vitro-grown *Impatiens hawkerii* and *I. walleriana*, however, revealed the presence of five SOD isoforms: two slow-migrating MnSODs, one FeSOD and two Cu/ZnSODs with highest mobilities [27]. These five isoforms were present in both healthy plants and those infected with tomato spotted wilt virus. Significant differences in SOD isoform profiles between in vitro- and ex vitro-grown plants have also been shown for different potato cultivars [14]. The authors reported that the most important qualitative difference between ex vitro- and in vitro-grown potato plants was the presence of additional FeSOD and Cu/ZnSOD isoforms in plantlets grown in vitro. The expression of FeSOD only in in vitro-grown potato cultivars was discussed in terms of compensation for lower Cu/ZnSOD abundance and activity, probably due to lower Cu availability in vitro [14]. Additional studies are required to elucidate whether this could also be the case with in vitro and ex vitro-grown *I. walleriana.* In any case, our results corroborate the findings that the in vitro environment can substantially affect the profile of SODs, and, as discussed later, other antioxidative enzymes as well.

The cumulative activity of the three SOD isoforms did not change considerably in watered plants during the course of the experiment, so it was virtually the same on the 1st and 16th day with slight fluctuations in the activities of individual isoforms (Figure 5B). In stressed D plants, cumulative SOD activity gradually increased with the drought duration, reached the maximum level in severely stressed plants (T3), and then decreased to control level upon rehydration (T4). The activity of chloroplastic Cu/ZnSOD was strongly induced in response to drought, but the other two isoforms contributed as well (Figure 5B). The most prominent effect of SA was a significant reduction of total SOD activity in severely stressed plants (DS, T3) in comparison with untreated D plants. However, in other experimental plants, SA caused small (WS and DS at T1 and T2) or significant (WS at T3) induction of SOD activity (Figure 5B). In a stringently controlled in vitro environment, total SOD activity of *I. walleriana* shoots increased with increasing PEG concentration, whereas SA caused a reduction of SOD activity at all PEG concentrations; both PEG and SA showed clear dose-response effects on SOD activity [25].

In most studies related to the effects of SA on drought-stressed plants, drought alone increased total SOD activity, just like in *Impatiens*, while SA also increased SOD activity, for example in tomato [9], *Ctenanthe setosa* [10], wheat [48] and *Brassica napus* [50].La et al. [50] also found that the expression of Cu/ZnSOD and MnSOD were significantly up-regulated by SA pretreatment and/or drought. Our finding that chloroplastic Cu/ZnSOD is the most inducible isoform, which responds both to drought-and SA-signaling, can be related to the importance of chloroplasts as one of the main sources of ROS in water-stressed cells. Namely, impairment of photosynthetic machinery, particularly chloroplastic electron transport, and the consequent generation of ROS is a major consequence of drought stress [6]. Upregulation of chloroplastic Cu/ZnSOD isoform, which is localized mainly on the stromal face of the thylakoid membranes where photosystem I is located [13] also points tochloroplasts as the main source of superoxide in stressed *Impatiens* plants.

**Figure 6:**
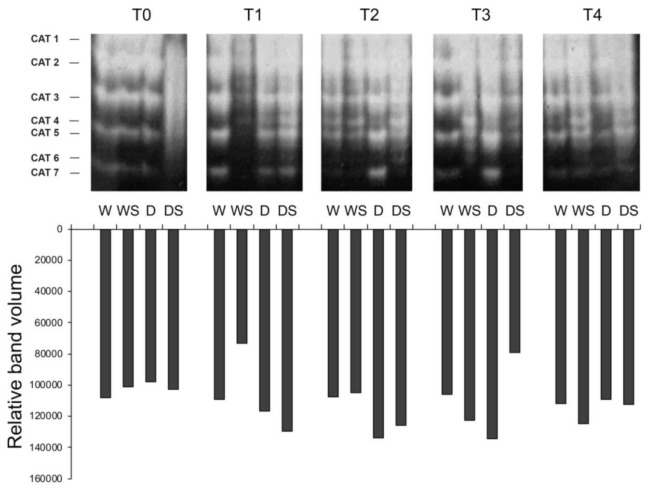
Zymograms of catalase (CAT) isoforms. Total soluble proteins isolated from leaves (35 μg protein/lane) of watered plants (W), drought-stressed plants (D), watered plants treated with SA (WS) or drought-stressed plants treated with SA (DS) were separated by native PAGE and assayed for CAT activity. T0–T4 are different sampling points (see Figure 1). Total CAT activities are represented by bars.

In-gel assays for catalase (CAT) activity revealed at least 7 possibly overlapping CAT activity bands in different treatments (Figure 6). The major bands or isoforms labeled as CAT1, CAT2, CAT3 and CAT5, as well as weak CAT7 activity, are present in all samples. Minor CAT4 activity is not present at T0, nor in most W samples (except weakly at T2), so it could be related to drought stress and/or SA treatment. Very faint CAT6 could be drought-inducible, since it appears in D and DS samples at all time points beyond T0 (Figure 6). Even though CAT activity profiles are complex, only three CAT genes have been identified in Angiosperm species analyzed so far [15]. The presence of multiple CAT activity bands was also observed in in vitro-grown *I. hawkerii* and *I. walleriana* [27], as well as in other species, which can be due to formation of heterotetramers composed of monomers encoded by different CAT genes, post-translational modifications and/or alternative splicing of the CAT transcripts [15]. Since neither drought stress, nor SA induce major changes in the CAT activity profiles, only total CAT activity is further discussed.

In unstressed (W) plants total CAT activity did not change over the course of the experiment (Figure 6). Drought stress caused a slight gradual increase in total CAT activity in D group of plants at time points T1–T3, followed by a slight decrease to the control (W) level upon rehydration at T4. Application of SA had little effect on the CAT activity, except decrease of CAT activity in WS plants immediately following SA application (T1) and in DS plants as compared to D plants after prolonged drought (T3, Figure 6). Total CAT activity in in vitro-grown *I. walleriana* increased in response to PEG and decreased in response to SA in a dose-dependent manner [25].

Drought stress impairs photosynthesis at many levels, including the reduction of CO_2_ influx due to stomatal closure and consequently CO_2_ assimilation, leading to enhanced metabolite flux through the photorespiratory pathway [6]. Oxidation of photorespiratory glycolate into glyoxylate in the peroxisomes generates H_2_O_2_ which is detoxified by the action of catalases. Thus, it is not surprising that increased CAT activity in response to drought stress has been reported for many species, e.g., in tomato [9], *Ctenanthe setosa* [10], *Celosia argentea* [24] and rice [49]. However, drought stress caused only a slight increase in CAT activity in *B. napus* [49], or had no effect in mustard [11]. Even though the activities of antioxidative enzymes are expected to be upregulated in response to drought, the fact that this is not so prominent in the case of CAT activities in *Impatiens*, rapeseed, and mustard can be explained in terms that leaf catalases can be highly expressed even under optimal conditions [15], as shown for *I. walleriana* (Figure 6). Regarding the effects of SA on CAT activities, the aforementioned species can be divided into three groups: (1) SA upregulated CAT activity in wheat, tomato, *C. setosa,* mustard, and rice; (2) SA downregulated CAT in *C. argentea* and *Impatiens;* and (3) response of CAT activity to SA was biphasic, as in *B. napus.* Namely, in SA-treated drought-stressed rapeseed, there was a sharp increase in CAT activity (10 days after the drought treatment) followed by a decrease in activity (15 days after drought treatment) [50]. The latest example highlights the importance of sampling time and explains differences in CAT activities in SA-treated *Impatiens* at different time points. Finally, it should be noted that SA can regulate CAT activity at least at two levels: at the transcriptional level, as shown for CAT induction in *B. napus* [50] and on the post-translational level, where SA can inhibit CAT enzyme by direct binding [22,23] which explains the different effects of SA on CAT activity.

**Figure 7:**
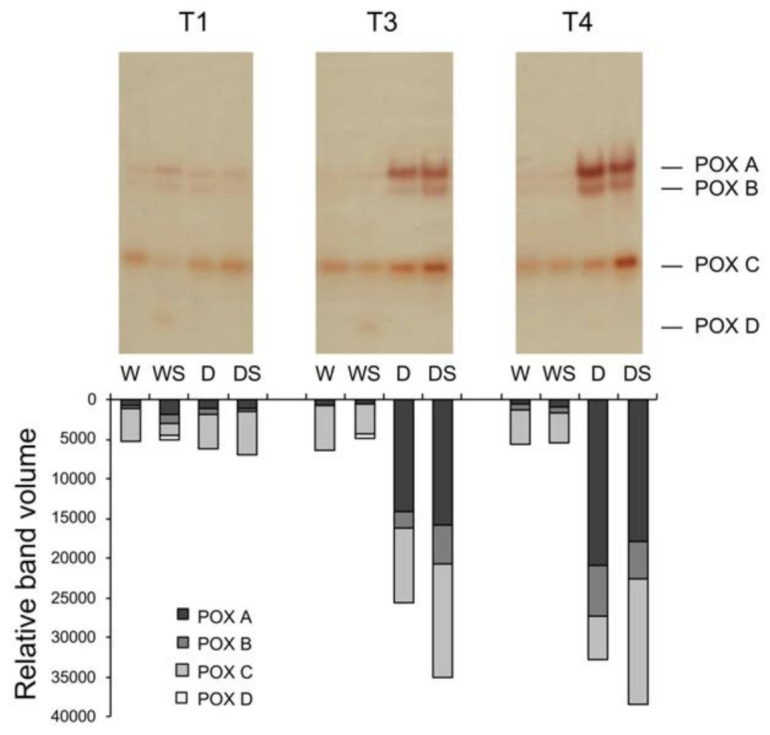
Zymograms of peroxidase (POX) isoforms. Total soluble proteins isolated from leaves (100 μg protein/lane) of watered plants (W), drought-stressed plants (D), watered plants treated with SA (WS) or drought-stressed plants treated with SA (DS) were separated by native PAGE and assayed for POX activity using guaiacol as a substrate. T0–T4 are different sampling points (see Figure 1). Densitometrically determined individual POX activities are represented by bars.

Peroxidase (POX) zymograms revealed activities of three major (POX A, B, and C) and one minor isoform (POX D, Figure 7). Since the POX isoform profile for ex vitro-grown plants presented here completely differs from POX profiles of in vitro-grown plants obtained by the same method [25,27], where as many as eight isoforms were detected under different treatments, the labeling of the isoforms does not correspond to previously published labeling. Watered plants showed low POX activity comprised primarily of POX C with faint POX A and B activities. Application of SA to watered plants did not change total POX activity, but induced very weak POX D, whose activity was not detected in other treatments (Figure 7). Severe drought stress significantly increased activities of all three major POX isoformsin comparison to W plants, particularly POX A (D group, T3), and this elevated activity further increased upon rehydration (T4). Application of SA to stressed plants significantly increased total POX and specifically POX C activities under severe stress (DS, T3) and upon rehydration (DS, T4) as compared to D group.

Drought response in many plant species involves remodeling of the cell wall—both loosening and tightening (including lignification), and some of these adjustments require ROS [51]. Among various enzymes involved in ROS production and metabolism at the cell surface and in the apoplast is a large family ofPOX enzymes, which may either use H_2_O_2_ to oxidize apoplastic substrates or reductants to produce O_2_^•-^ from O_2_; the generated superoxide may be then converted to H_2_O_2_ by the action of extracellular Cu/ZnSOD [51]. Due to a vast number of POX isoforms (e.g., 73 in *Arabidopsis*) and versatility of their functions [17], specific POX activities induced in response to some stress, hormone or other signal are rarely connected to a particular gene or physiological substrate, so their functions often remain unclear. One example of well-characterized POX isoform related to drought is *Arabidopsis AtPrx3,* whose overexpression in *A. thaliana* enhanced dehydration and salt tolerance, whereas it’s antisense suppression produced dehydration- and salt-sensitive phenotypes [52]. The actual functions of drought- and SA-inducible POX A, B, and C in *Impatiens* (Figure 7) are unknown, but they are obviously important components of drought response as well as in relief of water deficit.

### 3.4. Architecture of I. Walleriana Dehydrins and Their Expression in Response to Drought and SA

**Figure 8:**
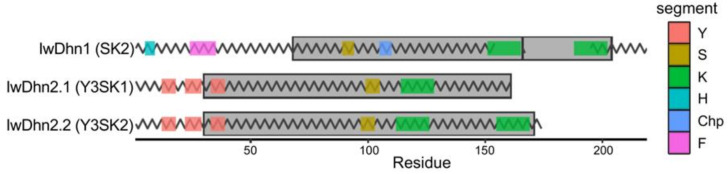
Schematic representation of three *I. walleriana* dehydrins. Grey boxes represent Dehydrin domains (pfam PF00257) according to hmmscan. The zig-zag lines represent disordered regions of proteins according to Espritz model with the default settings. Structural segments of dehydrins are shown as colored boxes. The drawing is to scale.

Dehydrins are composed of at least one family-defining K-segment, variable number of conserved Y-segments and a single S-segment, which are interspersed with regions that are not conserved and are generally termed φ-segments [18,20]. Lys-rich K-segment, present in all dehydrins, is usually [EKKGIMDKIKEKLPG], but none of its residues is absolutely conserved, so it is presented as [XKXGXX(D/E)KIK(D/E)KXPG] by consensus [20]. The K-segment is implicated in membrane binding [53,54]. The Y-segment, named after central Tyr residue is commonly [DEYGNP] motif [19], but it can vary within the [D(D/E/Q)(Y/H/F)GNP] consensus, with highly conserved residues underlined [20]. Y-segments are probably not involved in membrane binding and their role remains a mystery [18,20]. The S-segment is a stretch of 4–6 Ser in a row in the [LHR(S/T)GS4-6(S/D/E)(D/E)3] context [20]. Since dehydrins have been frequently extracted as phosphorylated forms, it has been shown that S-segment is a target for phosphorylation [19]. When phosphorylated, it can transfer dehydrins from the cytosol to the nucleus, even though some dehydrins without this segment also localize to the nucleus [20]. Depending on the arrangement of Y-, K- and S-segments, dehydrins are classified as one of five types: Kn, SKn, KnS, YnSKn, and YnKn [8,18,20]. Three dehydrin sequences were found in the *I. walleriana* transcriptome (GenBank accessions: MW219505, MW219506 and MW219507): one of the SK2 type named IwDhn1 and two with YnSKn composition: IwDhn2.1 (Y3SK1) and IwDhn2.2 (Y3SK2, Table 1 and Figure 8).

**Figure 9:**
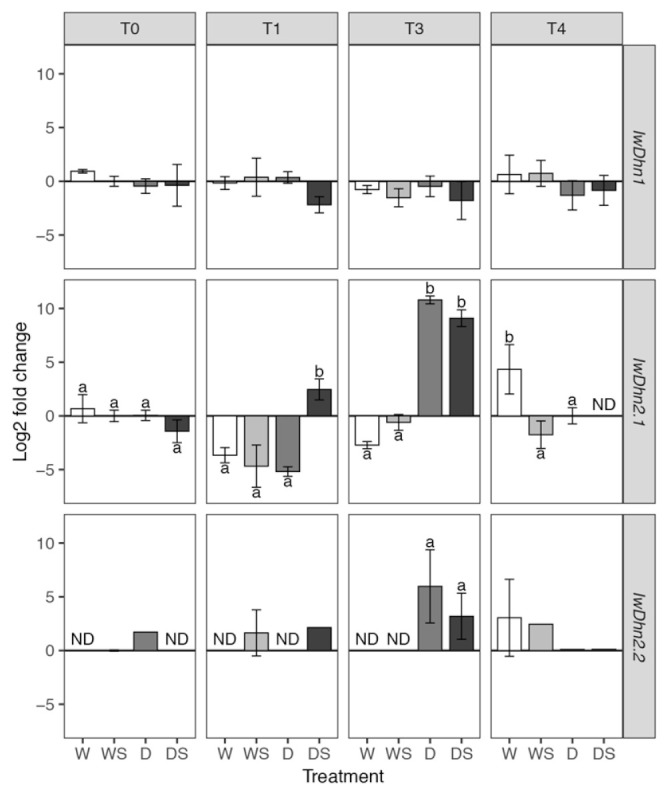
Relative expression of three *I. walleriana* dehydrins determined by quantitative real-time polymerase chain reaction (RT-qPCR). Groups of plants are labeled as W, WS, D and DS, while T0–T4 are sampling time points (see Figure 1). Means and standard deviations are shown for three biological replicates. ND—expression was not detected. IwDhn1 and IwDhn2.1 data was analyzed using factorial ANOVA: only drought stress had a slight but statistically significant effect on IwDhn1 relative expression (Table S8) so multiple comparisons between different treatments were not conducted. The effect of the three-way interaction dehydration:SA:time on the relative expression ofIwDhn2.1 was highly statistically significant (Table S9) so pairwise comparisons of estimated marginal means were conducted within each sampling time (T0–T4) using Tukey’s method and significant differences (*p* < 0.05) are indicated by a compact letter displayed above the bars. For IwDhn2.2 statistical comparison was conducted between D and DS treatments at T3 using Welch’s *t*-test.

IwDhn1 is a 24.5 KDa acidic protein expressed under normal conditions (T0), as well as in other treatments and time points with minor fluctuations (Figure 9). Three-way factorial ANOVA showed that only drought had a statistically significant but minor effect on *IwDhn1* relative expression, whereas the effects of SA, sampling time, or factor interactions were not significant (Table S8). D plants had slightly increased *IwDhn1* expression so that the level of *IwDhn1* mRNA was 1.43 (at T1) and 1.24 (at T3) folds higher when compared to the watered plants of the same age. Considering that *IwDhn1* is expressed in all treatments and time points, particularly under normal conditions in younger plants (T0), this suggests that this protein is important, but its expression profile implies that it is not crucial for drought stress protection. Indeed, comparison of dehydrins with different architectures and their abiotic stress regulators revealed that the SK2 type of dehydrins, such as IwDhn1, are not desiccation-inducible, but respond to cold and in some cases to salt stress, while some are constitutively expressed [8,18].

While the role of IwDhn1 is unknown, some clues can be drawn from the analysis of its sequence. Besides typical S- and K-segments, IwDhn1 also has H- and Chp-segments and a recently discovered F-segment (Table 1 and Figure 8). His-rich or H-segment is found in some dehydrins, like citrus cold-responsive dehydrin CuCOR15, with [HKGEHHSGDHH] motif able to bind metal ions (particularly Cu^2+^, but also Ni^2+^, Zn^2+^ and others), where His in H-X3-H and HH are involved in metal binding, as well as in DNA binding by intermediating Zn^2+^ [19,55]. The authors proposethat H-segment of CuCOR15 is involved in reducing metal toxicity, reducing Cu-promoted generation of ROS and DNA protection. It is questionable whether His-rich but quite different [HSHNH] motif found near the N-terminus of IwDhn1 (Figure 8) has similar properties. Charged peptide (ChP) consists of 1–2 polylysine segments, often preceded by Glu or Asp [18]. Thus the [EKKEKKKKKK] sequence present in IwDhn1 is a typical ChPn(Table 1). A similar [KKKKKKEKKK] motif, found in CuCOR15, was able to bind dsDNA, ssDNA and RNA in vitro, non-specifically and with relatively low affinity, in the presence of Zn^2+^ [19]. The authors suggested that this dehydrin, with its Chp segment, might protect nucleic acids during stress responses. Other proposed roles for Chp include nuclear targeting and chaperone activity [18]. It has recently been shown that the majority of SKn dehydrins contain additional F-segment, initially defined as [DRGLFDFLGKK] [54], but later redefined to a rather complex consensus given by [20], which was used to identify [ETQDRGILDFLK] in IwDhn1. The F-segment in SKn dehydrins, including IwDhn1, is localized N-terminal to S- and K-segments [54]. Secondary structure prediction models indicate that the F-segments may form amphipathic helices that could be involved in membrane or protein binding [53]. Considering the complex HFSChpK2 structure of IwDhn1 and the discussed possible roles of each of its segments, this dehydrin might be a multifunctional protein.

Unlike *IwDhn1*, *IwDhn2.1* and *IwDhn2.2* are strongly induced by drought (Figure 9). Expression of *IwDhn2.1* is induced~10^4^ fold in severely stressed plants (D, T3) in comparison to control plants of the same age. In the case of *IwDhn2.1,* the interaction of factors SA, drought and sampling time was highly significant (*p*< 0.001, according to three-way ANOVA, Table S9), meaning that SA had different effects on watered and stressed plants at different time points. SA induced *IwDhn2.1* expression in DS plants (as compared to D group) immediately following application (T1), but decreased its expression in severely stressed DS plants at T3 as compared to D plants (although this effect was not statistically significant). The expression of *IwDhn2.2* was undetectable or very low at most time points, except in severely stressed plants (D, T3), where *IwDhn2.2* was highly expressed. SA application lowered *IwDhn2.2* expression of stressed plants at T3, but this was not statistically significant (Figure 9). Expression of both *IwDhn2.1* and *IwDhn2.2* in drought-stressed plants was completely inhibited by rehydration (T4). These two dehydrins share not only similar expression profiles, but also the Y3SKn architecture (Table 1) and are 93.2% identical at the amino acid level and 89.9% at the nucleotide level (Table S1).Comparison of dehydrins with different architecture and stress types that induce their expression revealed that YnSkn dehydrins are commonly induced by desiccation and salt stress, suggesting a role for Y-segment in desiccation protection [18]. The main feature of IwDhn2.1 and IwDhn2.2 is the presence of three N-proximal copies of the Y-segment (Figure 8), which is relatively common in dehydrins [20]. Of these, Y1- and Y2-segments in both *I. walleriana* dehydrins are typical [DEYGNP] motifs [19], while Y3 is [DQYGNP] (Table 1). Finally, the K2-segment in IwDhn2.2 is flanked by His, a feature found in many dehydrins, that has an effect on membrane binding [53].

SA may, directly or indirectly, induce certain genes involved in protective mechanisms against biotic and abiotic stresses, including some dehydrins [23], which may explain the small induction of *IwDhn2.1* following SA application. On the other hand, the fact that SA application slightly decreased the expression of all three *Impatiens* dehydrins in DS plants relative to D plants at T3 (even though none of these changes were statistically significant) is more likely due to previously discussed SA drought-ameliorating effects, so that dehydrins are not needed as much as in D plants. An alternative explanation that SA directly downregulates transcription of dehydrins is possible but unlikely. Sun et al. [56] also reported that SA decreased the levels of four dehydrin-like proteins induced by water stress in Tibetan hulless barley seedlings.

## 4. Conclusions

Drought stress has adverse effects on potted *I. walleriana*, including wilting, floral abortion, reduction of RWC and increased lipid peroxidation as a consequence of oxidative stress. To cope with drought, *Impatiens* plants accumulate Pro and induce chloroplastic Cu/ZnSOD and two POX isoforms. The most remarkable drought response, however, is extraordinary induction of dehydrins *IwDhn2.1* and *IwDhn2.2*. Rehydration restored RWC, Pro level, Cu/ZnSOD activity and dehydrins expression in drought-stressed plants approximately to the values of watered plants. Foliar application of 2 mM SA had ameliorating effects on plants exposed to drought, including prevention of wilting, preservation of RWC, increased Pro accumulation, modulation of antioxidative activities and a remarkable decrease of lipid peroxidation. Unfortunately, SA did not show an effect on flowers preservation during drought, but even though flower abortion is a commercially undesired effect, it is one of the defense mechanisms ofa plant. Comparison of current results with previously studied responses of in vitro-grown *I. walleriana* revealed many similarities, yet also some differences, particularly in antioxidative enzyme isoform profiles, but they corroborated findings that SA can be safely applied to improve drought tolerance in *Impatiens* with no negative effects.

## Figures and Tables

**Table 1:** Characteristics of dehydrins found in *I. walleriana* transcriptome. The actual position of each segment is provided in Table S1.

Dehydrin	IwDhn1	IwDhn2.1	IwDhn2.2
Accession	MW219505	MW219506	MW219507
Length (bp)	960	537	863
Amino acids	219	161	174
MW (Da)	24487	16716	18198
pI	4.97	6.19	7.92
Architecture	SK2	Y3SK1	Y3SK2
Y1	/	DEYGNP	DEYGNP
Y2	/	DEYGNP	DEYGNP
Y3	/	DQYGNP	DQYGNP
S	LHSDSSSSSSDEEE	LRRSGSSSSSSSEDD	LRRSGSSSSSSSEDD
K1	EKKGFLEKIKEKLPG	KKGLKEKIKEKLPG	KKGLKEKIKEKLPG
K2	EKKGILEKIKEKLPG	/	(H)EKKGIIDKIKDKLPG(SH)
H	HSHNH	/	/
F	ETQDRGILDFLK	/	/
Chp	EKKEKKKKKK	/	/

## Supplementary Materials

The following are available online at https://www.mdpi.com/2223-7747/9/11/1589/s1, Table S1. Excel file containing transcriptome and dehydrins sequences details. Supplement 2. Table S2: Analysis of deviance table for flower and bud data modeled using Poisson regression with log link, Table S3: Analysis of deviance table for bud data modeled using Poisson regression with log link, Table S4: Analysis of deviance table for flower data modeled using Poisson regression with log link, Figure S1: Number of A—flower buds and B—flowers developed per plant, Table S2.4: Analysis of variance table (type I SS) for RWC, Table S6: Analysis of variance table (type I SS) for MDA, Table S7: Analysis of variance table (type I SS) for proline, Table S8: Analysis of variance table (type I SS) for IwDhn1, Table S9: Analysis of variance table (type I SS) for IwDhn2.1.
